# P-2217. Effect of Gut Bacterial Diversity on Mortality in Patients with Candidemia

**DOI:** 10.1093/ofid/ofae631.2371

**Published:** 2025-01-29

**Authors:** Young Kyung Yoon, Seung Min Park, Soo Hyun Park, Jin Woong Suh, Jeong Yeon Kim, Jang Wook Sohn

**Affiliations:** Korea University College of Medicine, Seoul, Seoul-t'ukpyolsi, Republic of Korea; Korea University College of Medicine, Seoul, Seoul-t'ukpyolsi, Republic of Korea; Korea University College of Medicine, Seoul, Seoul-t'ukpyolsi, Republic of Korea; Korea University College of Medicine, Seoul, Seoul-t'ukpyolsi, Republic of Korea; Korea University College of Medicine, Seoul, Seoul-t'ukpyolsi, Republic of Korea; Korea University College of Medicine, Seoul, Seoul-t'ukpyolsi, Republic of Korea

## Abstract

**Background:**

The gut microbiota plays an important role in defense against infectious diseases. However, only a limited number of clinical studies have investigated the microbiome in patients with candidemia. The aims of this study were to determine the nature of intestinal microbiome and to investigate the effects of gut microbiota on mortality in patients with candidemia.

**Methods:**

This pilot study was conducted at a 1048-bed university-affiliated hospital in Seoul, South Korea, from November 2022 to February 2024. The study included adult patients (≥18 years old) diagnosed with candidemia. Total DNA was isolated from stool, and high-throughput sequencing was performed. Clinical data were collected from patient medical records. Multivariate logistic regression was used to identify the predictors of in-hospital mortality.

**Results:**

A total of 55 patients were identified. The overall in-hospital mortality rate was 41.8%. The most commonly isolated *Candida* species was *Candida albicans* (36.4%), followed by *Candida tropicalis* (34.5%), *Candida parapsilosis* (18.2%) and *Candida glabrata* (7.3%). The most predominant bacteria species in their intestinal microbiome were *Enterococcus* species followed by Enterobacteriacea groups. The median Shannon diversity index [median (interquartile ranges); 2.4 (1.5-2.8) vs 1.6 (1.0-2.1), *P* = 0.015] and abundance-based coverage estimator (ACE) richness index [71.3 (39.3-97.7) vs 32.2 (15.6-61.2), *P* = 0.021] of the gut microbiota were significantly higher in survivors than in non-survivors. In a multivariate Cox regression analysis, septic shock [adjusted hazard ratio (HR) 8.8, 95% confidence interval (CI) 1.2-62.0, *P* = 0.030] and Shannon diversity index (adjusted HR 0.3, 95% CI 0.1-0.7, *P* = 0.012) were independently associated with the in-hospital mortality.

**Conclusion:**

Our findings suggested that low gut bacterial diversity was independently associated with increased mortality in patients with candidemia. Exploring the variation of microbiome will potentially reveal new perspectives for the prevention and treatment of candidemia in the future.

**Disclosures:**

All Authors: No reported disclosures

